# A Clinical Contribution to Treatment of Urethral Stricture

**Published:** 1882-12

**Authors:** Frank Dudley Beane

**Affiliations:** New York City


### Clinical Reports.

### A Clinical Contribution to Treatment of Urethral Stricture.

By Frank Dudley Beane, A. M., M. D., of New York City.

W. J. L., aged 52 years, American, clerk, bilio-nervo-sanguine temperament; general health had always been good. First (and last) catarrh, virulent, urethræ in 1844, degenerating into gleet, which lasted for years. In 1851 suffered a severe contusion of the perinæum by being thrown upon the pummel of his saddle. No blood or difficulty in urinating followed, but an abscess formed, which was opened by Dr. Gurdon Buck, who kept a silver catheter in the bladder till the fistula was nearly healed (two weeks). He then dilated the strictures, which the patient had treated by the use of bougies for several years. Finding them resilient, the Doctor performed internal urethrotomy, and followed this up with dilatation to No. 10 (English gauge), this calibre being maintained for years by patient. Then came neglect of bougies, so that in 1867 the strictures were as small as No. 2 or 3 (English). At this time Dr. Buck performed Holt's operation, because patient desired immediate "full size" (?) dilatation, having but one day in the city. No. 10 (English) again used for years, till, through neglect again, the patient found himself suffering from contraction to No. 2 (English), considerable vesical catarrhal inflammation, and the symptoms below enumerated. Two days prior to calling me he had attempted to introduce a small bougie, but, instead of passing into the bladder in usual way, he felt it suddenly move on (without "grasping"), causing very sharp pain, and withdrawal followed by a drop or two of blood. This was in April, 1875, when he first consulted me, his condition at that time being as follows: Very frequent and painful micturition, almost constant pain in the perinæum, pain in the back, loins and abdomen, urine varied in quantity and appearance—always more or less cloudy—stream very minute, restlessness, slight feverishness, loss of appetite, incapacity for business. Detected two strictures, one at 4⅛ inches, the other at 5 3-16 inches, former = calibre No. 5, latter = calibre No. 6,* both tunnel-like, irritable, severely resilient.

At once put him upon morphia (for pain), occasional doses of belladonna, pareira brava, and continuous dilatation, beginning with catheter No. 9. Nourishing diet, including plenty of milk and broths. At the beginning of the fifth day of treatment a semi-circular indurated, painful swelling appeared to the left of where the scrotum joins the perinæum. At this time dilatation had been advanced to No. 16 bougie olivaire, which passed with difficulty and pain, tightly grasped; poultices; swelling increased, accompanied by decided chills; urine had to be drawn with catheter, great vesical tenesmus; was given potassa acetate and sodæ bicarb. several times daily, in addition to the morphia. Two and a half days later I opened the perinæal abscess under ether, allowing some f℥ij of highly offensive, greenish-yellow, pus, slightly streaked with blood, to escape; carbolized dressings; great general relief; urine drawn off with catheter for 24 hours. On following day passed No. 13 bougie olivaire, followed by catheter No. 11, drawing off ⅔ pt. urine.

Simple dilatation thereafter continued. Urine passed *per perinæum* two days later. Four days later the wound had almost completely filled up, only a few drops of urine passing through the fistula. One week later fistula completely closed. Eleven days subsequent, dilatation had reached No. 16 bougie olivaire. Passed urine six to eight times a day in quite a fair stream.

During treatment by simple dilatation patient had several *attacks of chills, followed by fever, this succeeded by sweating* (regular ague paroxysms) to which he had been subject for several years. Plenty of covering, hot drinks and absolute rest relieved these attacks, but quinia did not prevent them. These attacks were undoubtedly due to reflex action propagated from the strictures.

* French gauge always referred to in these pages.

On June 11th, following (one month and sixteen days from commencement of treatment), I was able to pass steel sound No. 17 for the first time. This was passed every four or five days till July 17th, when No. 18 went into the bladder. The use of this instrument was continued every six or seven days till the following December, afterward every two weeks, for a short time. Patient instructed to use it regularly every twelve to fourteen days, which was done until May, 1877, when I lost sight of him. He had remained in excellent general health since July, 1875, the bladder not emptying itself oftener than five to seven times daily; urine normal excepting the presence of a small amount of pus. No operative measures desired.

On Aug. 3, 1880, the patient again presented himself for treatment. His history since 1877 was: Business embarrassment, neglect of dilatation, quite a long sojourn out West, where he fell seriously ill from intermittent fever and his urinary troubles; returned home thoroughly broken in health, treatment (very meagre, simple dilatation and washing out bladder through a new fistula) at the hands of a Brooklyn physician. Worn out in body and mind, he again presented himself to me in the following condition: Great loss of flesh, cadaveric-sallow skin, almost complete loss of appetite, bowels irregular, general strength greatly reduced. A fistula *in perinæo* at a new site, through which nearly all the urine passes—only guttatim—through the urethra; pain and smarting in fistula; urine loaded with pus and phosphates; albumen (due mostly to pus); no casts; voided every one and a half to three hours. Occasionally, as of old, only more frequently, has "ague attacks." Quinia (gr. x: xv, daily), iron, pareira brava, claret wine, etc., as general treatment. Simple dilatation by bougies, beginning with No. 3. By introducing the bougies every one, two or three days, I was enabled to pass No. 12 at the end of two and a half months, but the strictures were so resilient and irritable

14

that there was but little result obtained as far as voiding urine *per urethram.**

During the latter part of October substituted ext. cannab. ind. (gr. ij : viij, daily) for morphia, and with good effect.

On November 16th he began upon quinia, ʒss daily, in three doses. On November 21st commenced continuous dilatation, beginning with catheter No. 10 and ending with No. 22 on December 1st. After the second day, catheters introduced under ether, so terribly irritable and tender the strictures. After removal of each catheter a prolonged warm sitz-bath given.

Examination of urine on December 6th found it acid, 1016, abundance of pus, albumen, phosphates in excess, otherwise normal.

On and after Dec. 4th, daily introduction of bougies Nos. 18 and 20; always grasped as in a vise; ether invariably given. Following these introductions, a minute stream *per urethram* for the first micturition, afterward only *guttatim.* Urine voided freely and almost painlessly through fistula. On Nov. 27th, began washing out the bladder, through catheter, passed *per urethram*, daily, with sol. potass. permang. (gr. i to f℥i). This treatment continued, at intervals of two to five days, without any material improvement till Dec. 20th, when a deep perinæal swelling showed itself a little posterior to the existing fistula. On January 1, 1881, it had approached the latter, fluctuation being easily detected, though situated deeply, I made a free incision and let out quite a quantity (perhaps f℥ij) of ill-smelling pus and blood. No urine found its way through this opening, which healed thoroughly in three weeks; antiseptic syringing and dressing. On the 7th of January (six days after above operation) soreness, succeeded by swelling, appeared in the region of the "membranous" urethra, pushing forward and downward on the right side toward the testicle, involving the scrotum,

* During the second week in November the strictures had become so irritable and resilient that only No. 4 could be passed, even this giving great pain. Scarcely any urine passed *per urethram*, and the fistula had become so much closed and irritable that considerable tenesmus and pain accompanied urination, which occurred every one to one and a half hours during the day, every two hours during the night. His general health had become greatly deteriorated by pain, loss of sleep and appetite, etc.

gradually acquiring an elongated shape, lying to the outer side of the testicle, its base at the junction of the ischium and pubes (right), its apex at the lowermost point of the right scrotum. Four days later I aspirated about fʒvj of very thick, ill-smelling pus from the tumor, as temporary relief, freely incising it eight days later, letting out f℥ij bloody pus and about f℥i bloody urine. Thereafter urine flowed also through this new fistula as well as the other.

The quinia—ʒss daily—nourishing, digestible food, milk punches, local syringing, occasional hip-baths, passage of bougies was continued since Nov. 7, 1880, and in addition began upon a mixture of iron and strychnia. Morphia occasionally taken for excessive pain or irritability. Feb. 11th, scroto-perinæal wound almost healed; gives exit still to urine. Considerable induration of scrotal tissues on right side, and upward at the base of the original swelling. Patient improved a little in general health; can exercise some in open air.

March 25th.—Urine same physical conditions as last reported. Average urination, three hours; small stream through each fistula; about size of No. 8 bougie through urethra. Decided decrease in local induration. So far this month has been taking only ℈i quinia daily.

Explained to patient the danger to which he is exposed from this constant pressure upon the bladder, and indirectly upon the kidneys, as well as the *reflex* influence of the strictures upon the latter organs, dilating upon the importance of not longer relying upon palliative but at once resorting to operative measures, which will free him from suffering, ill-health, and the great danger which constantly menaces him. A few days later he communicated to me his decision, namely, to undergo such preparatory treatment as I thought best prior to operating. To use his own words: "I would rather die than live in this condition; I will take tne chances;" which, I assured him, were *decidedly* in his favor.

April 10th.—General as well as local improvement. Have continued use of bougie No. 18 every three to five days (always under ether), as well as tonic treatment above indicated. Urine cloudy, "yellow," acid, 1012, abundance of pus, 1-24 (vol.) albumen (due to dissolved pus, in great part). To have warm hip-bath this evening. To-morrow morn (8½ o'clock) to have quinia (ʒss), morph. (gr. ss), and digitalis (♏ viij).

April 11th, 10 A. M.—Pulse 96 (nervous excitement); temp. (tongue) 97.8° F.

Dr. B. W. Dyer assisting, patient under ether (one hour consumed in anæsthetizing, on account of frequent suspension of respiration, requiring gr. 1-60 strychnia hypodermically), I divided all the strictures but the last three to No. 34, using Otis' dilating—urethratome, large blade, and the meatus to No. 35 with meatatome. [On Dec. 7, 1880, I had again made a thorough exploration of the urethra, finding strictures at

| *Location.* | *Calibre.* | *Form.* | *Character.* |
|---|---|---|---|
| 1⅝ inch, | No. 13, | Annular, | Dilatable. |
| 2⅛ to 2¼ " | " 14, | Tunnel, | Gristly, highly resilient. |
| 2⅝ " | " 14, | Annular, | Fibrous. |
| 3 " | " 15, | " | " |
| 3¾ " | " 14, | " | " |
| 4⅛ to 4⅜ " | " 13, | Tunnel, | Gristly, highly resilient. |
| 5¼ " | " 13, | Annular, | Dilatable. |

Size of penis = 3¼ inches; corresponding size urethra = No. 32.] Very moderate bleeding; at once controlled by pressure and sponging with cold water. Carbolized plug within meatus; carbolized oil and absorbent-cotton dressing. To take quinia (℈i), morphia (gr. ⅓), and digitalis (tinct. = ♏ v) at 1 P. M. At 5 P. M. take ♏ v tr. digitalis.

Recovered well from ether. Had a severe chill from 4.43 to 5.15 P. M., having urinated at 4 P. M., very moderate bleeding, but pain quite severe. Had quinia, morphia and digitalis at once, with additional morphia for pain. 9 P. M.: Has urinated twice since 4 P. M., less pain, but moderate bleeding. Very comfortable. Pulse 84, full, strong; temp. 98.2° F.

April 12th.—Good night; urinates about every two hours; bloody till last time this morning. Urine: acid, 1016, full of pus, no blood, 1/4 albumen (renal congestion); no head or other bad symptoms. Quinia, morph. and digitalis treatment, with cream, milk and Vichy.

April 13th.—Slept well; urine: acid, 1020, pus, 1-10 albumen. 10 P. M.: pulse 72, temp. 97.8° F. Thoroughly under digitalis, (constant nausea).

14th.—Doing excellently; takes plenty of milk and cream. Medicaments as before, but less digitalis. No nausea. Urine: acid, 1012, pus, 1-15 albumen.

15th.—To-day (96 hours after operation) for first time passed sound (straight) No. 33 down to stricture at 3¾ in. with ease (under ether). Very moderate bleeding.

23d.—Sound passed every day but yesterday. Appetite excellent and general condition good. For the twelve days since the operation the patient has taken: Quinia, grs. 675; morphia, 11⅔ grs.; tr. digitalis, ♍ 136. Slept well last night. Bright and cheerful this morning. Urine: 1-9 (vol.) albumen.

At this 8 A. M. had quinia, gr. xv, cinchoniæ sulph., gr. xv, morphia, gr. ⅓, and digitalis, ♍ viij.

At 10.35 began etherization; stoppage of respiration as before, hypodermic of strychnia gr. 1-60, anæsthetization complete at 11.20. Dr. B. W. Dyer again assisting, divided all but the stricture at 5¼ inches thoroughly to No. 33.

Very little hemorrhage. Bougie á boule No. 32 passes easily down to 5¼ inches; No. 21 bougie á boule passes contraction at this point. Carbolized instruments, sponges, and dressing, as at former operation. On recovering from ether, pain so great as to require hypodermic of gr. ss morphia.

Same treatment as before pursued, only pushed the digitalis more rapidly and to utmost physiological effect.

26th.—Introduced straight sound daily to 3¾ inches. To-day the same, and No. 30 curved sound into bladder. Ether. Very little bleeding. Urine: only a trace of albumen. No chill after this operation.

27th.—Sound No. 33 to 3¾ inches; sound No. 32 into bladder (ether). Only *drops* pass through the fistulæ; large urethral stream. Good appetite. Sleeps well. No suffering whatever. Has had, on average, each day, quinia (or cinchoniæ sulph.) gr. xlv; tr. digitalis ♍ x; morph. gr. ⅔.

May 2d.—Unretarded progress, sounds passed daily, always under ether. Eats and sleeps well. Very cheerful. Urine passes in excellent stream, *none through fistulæ.* To-day and henceforth sound No. 32 only. Has averaged, daily: cinchon. sulph. ʒi; morph. gr. ss; tr. digitalis, ♍ xiv. Sat up yesterday; out in coach to-day.

16th.—Urine: only trace of albumen. Went out walking on the 4th inst. and daily since. Sound passed every day or alternate days; ether. Cinchon., sulph. only, ʒss daily. Digitalis and morphia discontinued. On 7th began upon acid benzoic., ℈i thrice daily. On 11th inst. took too much exercise in park, which caused a temporary elevation of temperature, *malaise*, headache. Increased doses of quinia (substituted for cinch. sulph.) removed symptoms. All local induration gone some days since.

The highest pulse following the first operation .............88.
" " " " " second operation...........76.
" " temperature following the first operation........98.2° F.
" " " " " second operation .....98.3° F.

20th.— Discontinue ammon. benzoas, substitute gtt. xv ol. eucalypt. three times daily.

During June sound passed every four or five days.
" July sound passed every week to ten days.
" August sound passed every two weeks.
" September sound passed twice.
" October sound passed twice.

A slight accumulation of pus found in track of second fistula, which I opened on Oct. 21st, letting out a few drops of pus. Syringing and cleanliness.

Sound passed twice during November, when remaining fistula completely healed up. A week later the scrotum and perinæum normally soft. Sound passed at intervals of four to five weeks since, always under ether. Discontinued ol. eucalyp. in July; no special benefit. Patient has enjoyed most complete health since a year ago, having an excellent complexion, good appetite and sleep, gained in flesh, and able to enjoy life with his fellow-beings. Urinates about six times daily; splendid stream. Urine: acid, normal sp. gr., "yellow," very little pus, more mucus and epithelium, very faintest trace albumen (due to pus).

Sound No. 32 always passes through the urethra anterior to remaining stricture with the most perfect ease. Stricture at 5¼ in. still shows contractility, so I am indisposed to allow a longer interval between the introductions than five weeks, at least at present. Patient takes long walks, attends to his business with the utmost regularity, in fine, is in the enjoyment of most robust health for a man of his years.

Remarks.—Inasmuch as this case more fully illustrates the wonderful effects of complete division of urethral strictures than ordinary cases met in practice, there are a few points connected herewith which seem to me to be worthy of special mention.

First, the cause was not only gonorrhœal, but traumatic as regards the contraction at 4⅛ to 4⅜ inches, which accounts for its highly cartilaginous and resilient nature.

Right here it might be well to call attention to the utter inefficiency of the incomplete division of the existing strictures by Dr. Buck, since methodically frequent use of bougies was required to maintain even imperfect patency of the canal. Worse still in its ulterior effects was Holt's operation, which undoubtedly greatly added to the irritability and resiliency.

The patient's own attempt at passing a bougie resulted in making a false passage, through which a few drops of urine became extravasated, giving rise to the first abscess and fistula, which so readily healed under appropriate dilatation.

On account of disinclination to submit to other than the simplest measures continuous, followed by simple dilatation, was employed as holding out the best prospect of such relief as would be permanent, if the requisite of such treatment was complied with, namely, regular use of sound at stated, short intervals. The reflex vaso-motor effects of a damaged urethra, in the way of "ague-attacks," were especially remarkable. Half-drachm doses of potassium bromide frequently had the effect of preventing or cutting them short.

When the patient presented himself in August, 1880, no operative measures were to be entertained. The desideratum was to give as much local relief as possible by the gentlest means, and to build up the general health so that he would be prepared for a radical cure of his strictures (saving the last). This treatment was very slow and tedious, and marked by most discouraging complications.

If evidence of the total—aye disastrous—inefficacy of dilatation in any form were needed to induce surgeons to abandon it in all but the simplest cases of "penile" contractions (of large calibre) of the urethra, it seems to me that cases like the present should furnish the missing link. Abscesses, fistulæ, constant pain, severe vesical catarrh, and indirect pressure upon the kidneys—surely 'tis a sad picture to look upon.

Behold, on the other hand, the almost miraculous relief afforded by an operation which restores the urethral canal to its normal calibre.

That Dr. Otis has given the profession an operation (and instrument) which, if rightly performed, is capable of absolutely curing "penile," urethral strictures will not be denied by those who have followed the old views and practices (notably those formerly taught by Sir Henry Thompson) and they those advocated by the New York reformer.

I can add nothing of interest to what has been better said by the distinguished professor in regard to the details and requirements necessary to the obtainance of perfect results.

I simply draw attention to the fact that in the case here reported the urethra anterior to 5¼ inches is as smooth and free from all tendency to recontraction as an undamaged urethra of a perfectly healthy man. Nor is this the result in a single case only; since I exposed the theory and practice marked out by Dr. Otis, this perfect result has been the rule in a series of many cases extending over a period of nearly four and a half years.

There are some points connected with preparatory and after-treatment in operating which I consider important in securing successful issues.

First, as regards preparatory treatment. I think it highly essential to the prevention of complications for the patient not only to be "built up," but to be thoroughly submitted to the physiological influence of quinia (or cinchonia) for at least three days prior to the operation. About one hour and a half previous to the latter I invariably have the patient take from gr. xv to xxx of quinia, gr. ¼ to ⅓ of morphia, and in cases where there is the faintest suspicion of previous or present disturbed renal function, a full dose of digitalis (tincture or infusion). This same dose to be repeated as soon after the operation as the state of the patient's stomach may allow. Nor do I end this prophylactic treatment here; in all but the simplest cases (meatotomy) I maintain a mild cinchonism (with or without morphia), for five to seven days. In cases of apprehended renal trouble I also give digitalis in doses to maintain mild physiological effects. Under this regimen my patients have no chills, fever (of any consequence), or other disagreeable complications (excepting the chill after the first operation in this case). I believe the notable absence of even a single bad symptom in this case was due to the special preparatory and after-treatment; indeed, I doubt not it warded off possible serious complications. A rule of paramount importance, in my opinion, is the non-introduction of a sound, or other meddling with the urethra, sooner than seventy-two, better ninety-six, hours after the operation.

If thorough division of the strictures have been performed, there will be no necessity for an earlier use of the sound. Finally, let us fully appreciate the benefits conferred by the operation upon this patient—re-establishment of robust health, removal of all danger to the kidneys, freedom from pain, and reduction of pus in the urine to almost *nil.*

There is but one regret, namely, that a removal of all contractions by division of the posterior stricture is inexpedient by reason of the very considerable risk to be incurred.

Let us hope that ere long we may learn of a comparatively safe method of *completely* dividing strictures located at the "membranous" urethra. Divulsion I reject as of eventual injury, though, may be, of temporary good.

Electrolysis requires further investigation as a *curative* agent.

